# Characterization and Expression of Glutamate Dehydrogenase in Response to Acute Salinity Stress in the Chinese Mitten Crab, *Eriocheir sinensis*

**DOI:** 10.1371/journal.pone.0037316

**Published:** 2012-05-17

**Authors:** Yueru Wang, Erchao Li, Na Yu, Xiaodan Wang, Chunfang Cai, Boping Tang, Liqiao Chen, Alain Van Wormhoudt

**Affiliations:** 1 School of Life Science, East China Normal University, Shanghai, China; 2 School of Basic Medicine and Biological Science, Soochow University, Suzhou, China; 3 Jiangsu Provincial Key Laboratory of Coastal Wetland Bioresources and Environmental Protection, School of Basic Medicine and Biological Science, Yancheng Teachers University, Yancheng, China; 4 UMR5178, Station de Biologie Marine du Muséum National d'Histoire Naturelle, BP225, Concarneau, France; Karlsruhe Institute of Technology, Germany

## Abstract

**Background:**

Glutamate dehydrogenase (GDH) is a key enzyme for the synthesis and catabolism of glutamic acid, proline and alanine, which are important osmolytes in aquatic animals. However, the response of GDH gene expression to salinity alterations has not yet been determined in macro-crustacean species.

**Methodology/Principal Findings:**

GDH cDNA was isolated from *Eriocheir sinensis*. Then, GDH gene expression was analyzed in different tissues from normal crabs and the muscle of crabs following transfer from freshwater (control) directly to water with salinities of 16‰ and 30‰, respectively. Full-length GDH cDNA is 2,349 bp, consisting of a 76 bp 5′- untranslated region, a 1,695 bp open reading frame encoding 564 amino acids and a 578 bp 3′- untranslated region. *E. sinensis* GDH showed 64–90% identity with protein sequences of mammalian and crustacean species. Muscle was the dominant expression source among all tissues tested. Compared with the control, GDH expression significantly increased at 6 h in crabs transferred to 16‰ and 30‰ salinity, and GDH expression peaked at 48 h and 12 h, respectively, with levels approximately 7.9 and 8.5 fold higher than the control. The free amino acid (FAA) changes in muscle, under acute salinity stress (16‰ and 30‰ salinities), correlated with GDH expression levels. Total FAA content in the muscle, which was based on specific changes in arginine, proline, glycine, alanine, taurine, serine and glutamic acid, tended to increase in crabs following transfer to salt water. Among these, arginine, proline and alanine increased significantly during salinity acclimation and accounted for the highest proportion of total FAA.

**Conclusions:**

*E. sinensis* GDH is a conserved protein that serves important functions in controlling osmoregulation. We observed that higher GDH expression after ambient salinity increase led to higher FAA metabolism, especially the synthesis of glutamic acid, which increased the synthesis of proline and alanine to meet the demand of osmoregulation at hyperosmotic conditions.

## Introduction

Salinity is one of the most important factors influencing the physiological status of aquatic animals. Changes in ambient salinity are directly related to osmoregulation capacity [1], [2]. During acclimation to salinity, the main challenge for aquatic animals is to regulate their osmotic pressure to maintain normal life activities [3]. The Chinese mitten crab, *Eriocheir sinensis*, is a strong osmoregulator and has been used as a model species in a number of physiological investigations [4], [5]. *E. sinensis* can control its hemolymph composition at a near constant level over the complete range of salinities from seawater to brackish water and even freshwater [4], and *E. sinensis* can function equally well in freshwater or marine conditions [6]. Previous studies have primarily focused on the functions of the gills and gill epithelium [7], particularly the role of the sodium pump in active transport of ions across the gills [8], [9] and enzyme activity in relation to the effects of neuro-endocrine factors, such as dopamine and carbonic anhydrase, on ion transport through the gills [10], [11]. Rathmayer and Siebers (2001) reported on the mechanisms that control the balance of sodium and chloride ions in freshwater-acclimated mitten crabs [6]. Their results revealed that the ability of juvenile and adult *E. sinensis* to cope with salinity variations during their life cycles involves ontogenetic changes in their osmoregulatory capacity [5], [9]. However, the underlying mechanism of osmoregulation of *E. sinensis* has not been fully revealed.

Regulation of hemolymph osmotic pressure mainly depends on the permeability of water and ions, and changes in the content of osmotic pressure effectors. The hemolymph concentration of ions and free amino acids, accompanied by metabolites from the blood, account for most of the hemolymph osmotic pressure [12], [13]. Free amino acids play important roles in determining the cell volume and osmoregulatory processes of many organisms, particularly Mollusca and Crustacea, fish, amphibians and reptiles [14]. Intracellular accumulation of free amino acids is a common response of many organisms to changes in ambient salinity [15]–[18]. Free amino acids that accumulate in response to hyperosmotic stress are called compatible osmolytes because they can regulate their cell volume and stabilize cellular macromolecules [3]. In euryhaline crustaceans, free amino acids comprise the bulk of organic osmolytes accumulated in response to hyperosmotic stress. Previous studies indicate that proline, alanine, glutamic acid, glycine and taurine play important roles in the osmoregulation of crustaceans but also differ among species [19]. For *E. sinensis* adapted to seawater, amino acids are much more concentrated in the muscle than in the blood [20]. However, the main concentrated amino acids, proline, glycine, alanine, taurine and glutamic acid, are the same in both tissues. During adaptation from seawater, proline concentrations follow the same decreasing pattern as the other amino acids in the muscle [20]. Therefore, glutamate, a precursor to proline, has been considered a major regulatory checkpoint in the proline synthetic pathway for osmotic regulation [21].

Previous studies on other crustaceans suggest that the activities of key enzymes in the synthesis of amino acids are influenced by intracellular ions and can lead to a net accumulation of amino acid when these enzymes are up-regulated by salinity [22], [23]. Glutamate dehydrogenase (GDH) has been invoked as a potential control point for amino acids synthesis because it is involved in the production of glutamate from α-ketoglutarate. Transamination of pyruvate with glutamate produces alanine, while proline is synthesized from glutamate via a pyrroline-5-carboxylate intermediate. An increase in glutamate production via GDH could increase the syntheses of both proline and alanine. Therefore, GDH might play an important role in osmoregulation. The role of GDH in osmoregulation has been tested through the pathway of amino acid catabolism in various crustacean species [24], [25]. Despite the importance of GDH for osmoregulation, our understanding of the transcriptional regulation of this enzyme in crustaceans is very limited.

Li et al. (2009) reported two different GDH cDNA sequences (EU496492, AM076955) from *Litopenaeus vannamei*, and they found that these two cDNA were mainly expressed in muscle [26]. When *L. vannamei* were fed higher protein diets, they displayed higher GDH expression and activity [27]. However, Willet and Burton (2003) reported that GDH mRNA levels did not increase during hyperosmotic stress in *Tigriopus californicus* [21]. To date, no other information has been reported on the relationship between the GDH gene expression and acute salinity stress in macro-decapods, which are grown as an important economic species.

In this study, the full-length GDH cDNA sequence of *E. sinensis* was cloned and characterized. A quantitative real-time polymerase chain reaction assay was developed to analyze the relative GDH expression, and the expression profiles were determined in different tissues and in the muscle of crabs transferred from freshwater to water with 16‰ and 30‰ salinities. Changes in the free amino acid content of muscle were also analyzed to verify the hypothesis that GDH regulates amino acid content in response to salinity stress.

## Results

### Characterization of GDH cDNA from *E. sinensis*

**Figure 1:**
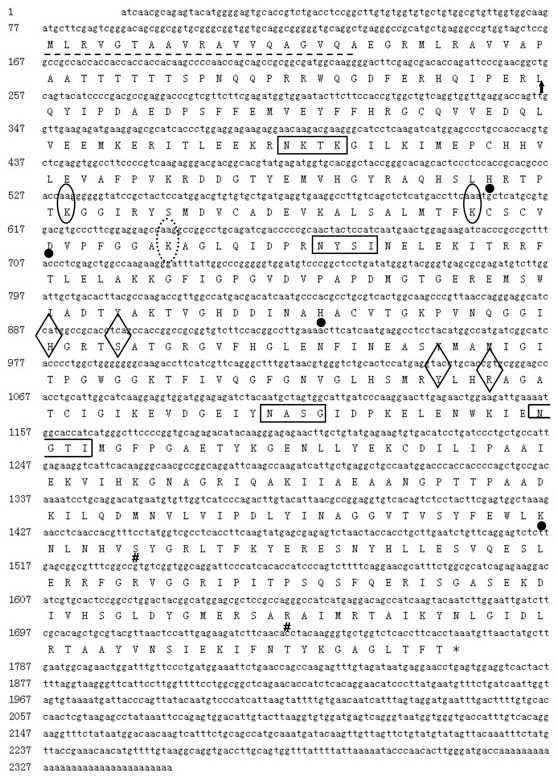
Complete cDNA and deduced amino acid sequences of GDH from *E. sinensis*. The signal peptide amino acid sequences are marked by a dashed black line. The cleavage site for the mitochondrial signal peptide is indicated by an arrow. The putative N-glycosylation sites are marked by black rectangles. The substrate binding sites, active site, NAD biding sites, GDP binding sites and ATP binding sites are annotated by a full line circle, a dotted line circle, a solid black dot, a diamond-shaped box and “**#**”, respectively. The asterisk (*) indicates the stop codon.

**Figure 2:**
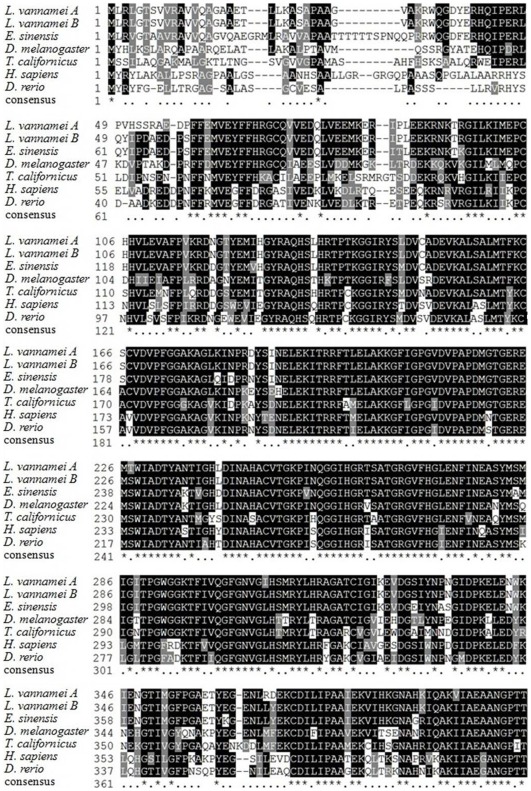
Amino acid alignment of GDH from *E. sinensis* with other species. Accession numbers for the GDH cDNA for each organism are as follows: *H. sapiens*, U08997; *D. rerio*, AY577003; *D. melanogaster*, Y11314; *T. californicus*, AY292656; *L. vannamei A*, AM076955; and *L. vannamei B*, EU496492. The shaded regions indicate identical residues, and other conserved, but not consensus amino acids, are shaded in grey.

The full-length GDH cDNA sequence cloned from *E. sinensis* was 2,349 bp long, contained a 1,695 bp ORF encoding a 564 amino acid protein, a 76 bp 5′UTR and a 578 bp 3′UTR (Figure 1), and was deposited in GenBank as JN628041. The deduced protein included a 19 amino acid signal peptide, four putative N-glycosylation sites, two substrate binding sites, an active site, four NAD biding sites, four GDP binding sites and two ATP binding sites (Figure 1). A putative mitochondrial signal peptide is likely present (human cleavage site indicated on alignment). Sequence comparison of the deduced GDH amino acid sequence showed 90% - 64% identity to that of *L. vannamei*, *Drosophila melanogaster*, *T. californicus*, *Danio rerio* and *Homo sapiens* (Figure 2).

**Figure 3:**
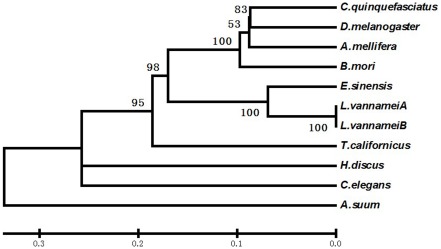
Neighbor-joining phylogenetic tree of GDH amino acid sequences of *E. sinensis* and other species. Accession numbers for each GDH homologs are as follows: *Culex quinquefasciatus*, XP001864850.1; *D. melanogaster*, Y11314; *Apis mellifera*. XP392776.2; *Bombyx mori*. NM001046780; *E. sinensis*, JN628041; *L. vannamei A*, AM076955; *L. vannamei B*, EU496492; *T. californicus*, AY292656; *Haliotis discus discus*, ABO26678.1; *Caenorhabditis elegans*, NP502267.1; and *Ascaris sum*, ADY44480.1.

A phylogenetic tree was constructed by the neighbor-joining method (Figure 3) using GDH homologs of *E. sinensis* and 10 other invertebrate species. *E. sinensis* GDH fell into the expected position with the sequences of GDH from other arthropods based on organism phylogeny and showed close evolutional relationship with *L. vannamei*.

### Tissue expression of GDH from *E. sinensis*

**Figure 4:**
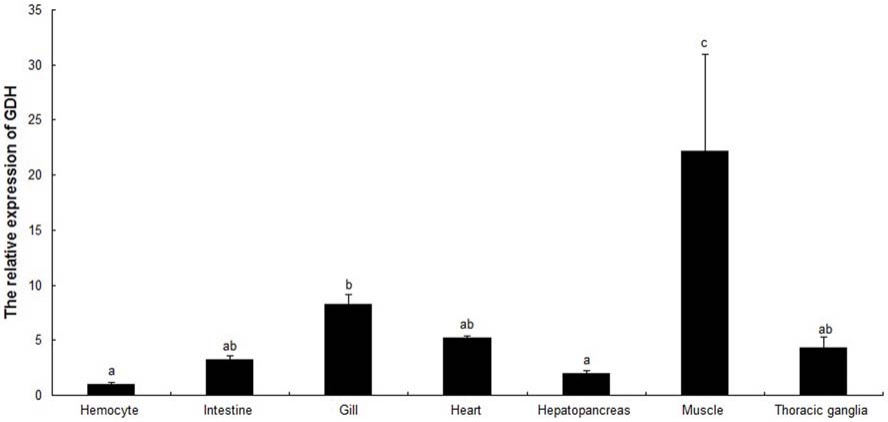
Relative GDH gene expression in different tissues of *E. sinensis*. β-actin was used as internal control, and different letters indicate *P*< 0.05.

GDH expression values in different tissues from *E. sinensis* are shown in Figure 4. GDH transcript expression was highest in muscle and significantly differed compared to other test tissues in this study (hemocytes, intestine, gill, heart, hepatopancreas and thoracic ganglia) (*P*<0.05). GDH cDNA transcript expression was second highest in the gill, which was significantly higher than in hepatopancreas and hemocytes (*P*<0.05), but no significant differences were observed when compared with the intestine, heart, and thoracic ganglia (*P*>0.05). The lowest GDH transcript expression values were observed in hemocytes and hepatopancreas.

### GDH expression in response to ambient salinity change

**Figure 5:**
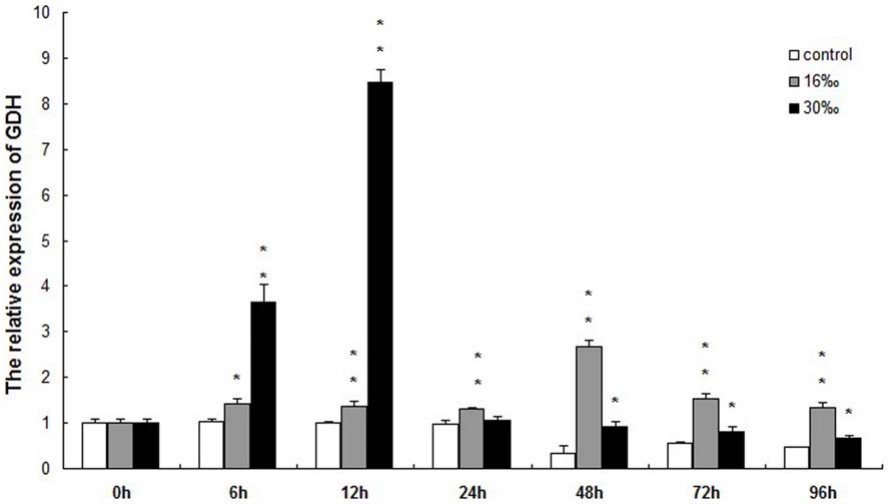
Relative GDH gene expression in E. sinensis muscle following acute salinity stress. β-actin was used as internal control, and “*” and “**” represent significant differences (*P*< 0.05 or *P*<0.01, respectively).

Figure 5 shows GDH gene expression over time in the muscle of *E. sinensis*, after transfer to water with 16‰ and 30‰ salinities, with freshwater serving as control. GDH transcript expression levels began to significantly increase at 6 h in both conditions of 16‰ (*P*<0.05) and 30‰ (*P*<0.01) salinity compared with control crabs in freshwater. GDH expression at 30‰ salinity continued to increase, peaked at 12 h (*P*<0.01), and gradually decreased from 24 h to 96 h. GDH expression was significantly higher (*P*<0.05) at all time points, except for 24 h. GDH expression in crabs at 16‰ salinity was significantly higher than the controls from 6 h to 96 h (*P*<0.05), peaked at 48 h (*P*<0.01) and gradually reduced from 72 h to 96 h.

### Free amino acid content in the muscle of *E. sinensis* changes under acute salinity stress

The free amino acid compositions in the muscle of *E. sinensis* at different sampling times are shown in Table 1. Total free amino acid content in the muscle began to increase significantly at both 16‰ and 30‰ salinity at 6 h as compared to the control crabs (*P*<0.05). Total free amino acid content at 30‰ salinity was reduced at 12 h, increased from 24 h to 96 h and peaked at 48 h. The total free amino acid content in crabs at 16‰ was not significantly altered (*P*>0.05) from 12 h to 96 h, except for the 72 h time point (*P*<0.05). Compared with the controls, the 16‰ and 30‰ salinity groups had significantly higher total free amino acids content at all time points (*P*<0.05), except for 72 h. The salinity stress response of essential free amino acids and non-essential free amino acids were similar to total free amino acids.

**Table 1:** Free amino acids in muscle of *E. sinensis* under acute salinity stress (mg/g wet tissue).

Free amino acids		Salinity							
	Time	contrl	16‰	30‰		Time	control	16‰	30‰
**EFAA**	0 h	2.432±0.152	2.432±0.152	2.432±0.152	**NEFAA**	0 h	4.442±0.227	4.442±0.227	4.442±0.227
	6 h	2.479±0.131^a^	2.98±0.124^b^	2.985±0.09^b^		6 h	4.281±0.213^a^	5.392±0.373^b^	5.576±0.316^b^
	12 h	3.095±0.099^a^	2.875±0.005^ab^	2.667±0.088^b^		12 h	4.686±0.177^ab^	5.361±0.338^a^	4.347±0.228^b^
	24 h	2.565±0.046^a^	2.839±0.018^a^	3.289±0.192^b^		24 h	5.021±0.338	5.275±0.013	5.628±0.19
	48 h	2.822±0.218	2.972±0.104	3.486±0.429		48 h	5.049±0.225^a^	5.427±0.249^ab^	6.574±0.672^b^
	72 h	2.681±0.076^a^	3.191±0.072^ab^	3.23±0.226^b^		72 h	4.803±0.436	5.588±0.015	5.61±0.2
	96 h	2.865±0.019^a^	3.098±0.003^b^	3.551±0.112^c^		96 h	4.822±0.438^a^	5.284±0.019^ab^	6.28±0.143^b^
Thr	0 h	0.132±0.018	0.132±0.018	0.132±0.018	Tau	0 h	0.199±0.001	0.199±0.001	0.199±0.001
	6 h	0.163±0.032	0.14±0.009	0.152±0.019		6 h	0.229±0.017^a^	0.418±0.012^b^	0.41±0.039^b^
	12 h	0.148±0.005^a^	0.151±0.006^a^	0.077±0.007^b^		12 h	0.449±0.116	0.385±0.051	0.411±0.02
	24 h	0.137±0.028	0.112±0.013	0.164±0.029		24 h	0.369±0.018^a^	0.243±0.021^b^	0.442±0.045^a^
	48 h	0.128±0.023	0.137±0.029	0.259±0.079		48 h	0.414±0.018^ab^	0.326±0.034^a^	0.453±0.044^b^
	72 h	0.071±0.005	0.113±0.016	0.103±0.035		72 h	0.275±0.068^a^	0.37±0.06^ab^	0.47±0.028^b^
	96 h	0.204±0.074	0.124±0.015	0.147±0.052		96 h	0.358±0.034^a^	0.306±0.026^a^	0.477±0.029^b^
Val	0 h	0.103±0.014	0.103±0.014	0.103±0.014	Asp	0 h	0.126±0.01	0.126±0.01	0.126±0.01
	6 h	0.085±0.023	0.106±0.012	0.126±0.011		6 h	0.214±0.045	0.188±0.033	0.15±0.051
	12 h	0.116±0.015^a^	0.109±0.003^a^	0.08±0.007^b^		12 h	0.006±0.000	0.22±0.068	0.255±0.137
	24 h	0.064±0.003	0.097±0.009	0.165±0.03		24 h	0.413±0.087	0.21±0.008	0.286±0.07
	48 h	0.109±0.031	0.109±0.012	0.18±0.054		48 h	0.342±0.056	0.287±0.042	0.257±0.091
	72 h	0.071±0.005	0.137±0.011	0.13±0.033		72 h	0.307±0.101	0.147±0.07	0.179±0.061
	96 h	0.064±0.001^a^	0.128±0.007^b^	0.244±0.019^c^		96 h	0.231±0.062	0.226±0.047	0.108±0.027
Met	0 h	0.061±0.009	0.061±0.009	0.061±0.009	Ser	0 h	0.283±0.035	0.283±0.035	0.283±0.035
	6 h	0.064±0.002	0.078±0.005	0.085±0.021		6 h	0.24±0.036^a^	0.385±0.023^b^	0.458±0.011^b^
	12 h	0.121±0.027	0.112±0.013	0.113±0.007		12 h	0.474±0.064	0.431±0.07	0.429±0.102
	24 h	0.083±0.007^a^	0.098±0.008^a^	0.138±0.007^b^		24 h	0.361±0.018^a^	0.337±0.02^a^	0.467±0.025^b^
	48 h	0.127±0.017	0.126±0.007	0.13±0.04		48 h	0.486±0.081	0.629±0.072	0.426±0.01
	72 h	0.136±0.034	0.1±0.015	0.209±0.056		72 h	0.376±0.032	0.413±0.053	0.4±0.013
	96 h	0.057±0.005^a^	0.156±0.008^a^	0.226±0.004^b^		96 h	0.412±0.109	0.385±0.028	0.509±0.081
Ile	0 h	0.099±0.017	0.099±0.017	0.099±0.017	Glu	0 h	0.256±0.028	0.256±0.028	0.256±0.028
	6 h	0.112±0.012^a^	0.117±0.003^ab^	0.15±0.009^b^		6 h	0.215±0.029^a^	0.233±0.026^a^	0.414±0.045^b^
	12 h	0.269±0.017	0.137±0.005	0.112±0.027		12 h	0.289±0.059	0.406±0.065	0.302±0.058
	24 h	0.101±0.01^a^	0.122±0.01^a^	0.227±0.01^b^		24 h	0.354±0.034	0.305±0.009	0.287±0.016
	48 h	0.125±0.031	0.134±0.01	0.216±0.048		48 h	0.318±0.038	0.315±0.029	0.411±0.027
	72 h	0.111±0.013	0.173±0.011	0.171±0.041		72 h	0.315±0.04	0.381±0.108	0.296±0.03
	96 h	0.134±0.012^a^	0.148±0.006^a^	0.229±0.008^b^		96 h	0.361±0.002	0.319±0.013	0.321±0.015
Leu	0 h	0.114±0.029	0.114±0.029	0.114±0.029	Gly	0 h	1.215±0.02	1.215±0.02	1.215±0.02
	6 h	0.115±0.028	0.091±0.032	0.135±0.018		6 h	1.168±0.018^a^	1.507±0.153^b^	1.337±0.031^ab^
	12 h	0.158±0.016^a^	0.14±0.002^a^	0.08±0.014^b^		12 h	1.322±0.06	1.336±0.006	1.298±0.022
	24 h	0.079±0.006^a^	0.141±0.01^ab^	0.201±0.033^b^		24 h	1.291±0.033	1.301±0.025	1.28±0.047
	48 h	0.152±0.058	0.146±0.017	0.212±0.052		48 h	1.343±0.057	1.296±0.022	1.391±0.045
	72 h	0.081±0.01^a^	0.183±0.018^b^	0.163±0.043^ab^		72 h	1.305±0.039	1.391±0.021	1.378±0.02
	96 h	0.093±0.001^a^	0.128±0.015^ab^	0.236±0.052^b^		96 h	1.36±0.02	1.38±0.025	1.348±0.023
Pro	0 h	0.035±0.009	0.035±0.009	0.035±0.009	Ala	0 h	1.147±0.035	1.147±0.035	1.147±0.035
	6 h	0.039±0.012	0.025±0.003	0.033±0.005		6 h	0.973±0.062	1.257±0.014	1.134±0.075
	12 h	0.033±0.01	0.031±0.001	0.031±0.003		12 h	1.196±0.083^a^	1.092±0.014^ab^	0.963±0.027^b^
	24 h	0.014±0.002^a^	0.031±0.003^a^	0.068±0.013^b^		24 h	1.034±0.045^a^	1.323±0.014^b^	1.146±0.037^a^
	48 h	0.035±0.014	0.037±0.008	0.079±0.021		48 h	1.065±0.017^a^	1.075±0.03^a^	1.192±0.014^b^
	72 h	0.026±0.002	0.061±0.021	0.077±0.027		72 h	1.09±0.062	1.148±0.054	1.299±0.067
	96 h	0.023±0.004^a^	0.032±0.012^a^	0.093±0.018^b^		96 h	1.286±0.047	1.25±0.079	1.297±0.063
His	0 h	0.128±0.021	0.128±0.021	0.128±0.021	Tyr	0 h	0.031±0.006	0.031±0.006	0.031±0.006
	6 h	0.11±0.009	0.132±0.002	0.124±0.012		6 h	0.037±0.006^a^	0.026±0.001^a^	0.04±0.004^b^
	12 h	0.139±0.027	0.141±0.005	0.137±0.006		12 h	0.035±0.009	0.032±0.002	0.043±0.004
	24 h	0.143±0.014^a^	0.153±0.004^ab^	0.199±0.019^b^		24 h	0.019±0.004^a^	0.04±0.003^a^	0.085±0.016^b^
	48 h	0.135±0.027	0.168±0.016	0.195±0.043		48 h	0.047±0.019	0.058±0.016	0.07±0.021
	72 h	0.152±0.011	0.172±0.003	0.211±0.053		72 h	0.036±0.006	0.074±0.031	0.105±0.048
	96 h	0.1±0.012^a^	0.156±0.005^b^	0.253±0.008^c^		96 h	0.034±0.011^a^	0.049±0.017^a^	0.118±0.013^b^
Lys	0 h	0.189±0.022	0.189±0.022	0.189±0.022	Pro	0 h	1.186±0.176	1.186±0.176	1.186±0.176
	6 h	0.205±0.015^a^	0.226±0.008^ab^	0.287±0.026^b^		6 h	1.206±0.112	1.379±0.147	1.632±0.323
	12 h	0.25±0.01	0.238±0.01	0.228±0.039		12 h	0.914±0.127^a^	1.46±0.122^b^	0.646±0.072^a^
	24 h	0.16±0.009^a^	0.275±0.015^b^	0.245±0.041^ab^		24 h	1.178±0.303	1.516±0.058	1.635±0.095
	48 h	0.187±0.013	0.262±0.052	0.283±0.064		48 h	1.035±0.141	1.442±0.172	2.374±0.699
	72 h	0.154±0.036	0.287±0.075	0.155±0.058		72 h	1.1±0.272	1.663±0.263	1.484±0.153
	96 h	0.245±0.031	0.221±0.011	0.149±0.035		96 h	0.78±0.241^a^	1.369±0.15^b^	2.101±0.632^c^
Arg	0 h	1.571±0.051	1.571±0.051	1.571±0.051	**TFAA**	0 h	6.874±0.091	6.874±0.091	6.874±0.091
	6 h	1.586±0.056^a^	2.064±0.138^b^	1.894±0.048^ab^		6 h	6.76±0.141^a^	8.372±0.495^b^	8.561±0.353^b^
	12 h	1.86±0.078	1.816±0.014	1.81±0.074		12 h	7.781±0.121^ab^	8.237±0.336^a^	7.014±0.261^b^
	24 h	1.784±0.023	1.808±0.017	1.882±0.047		24 h	7.586±0.294^a^	8.114±0.015^a^	8.916±0.138^b^
	48 h	1.826±0.034^a^	1.852±0.011^ab^	1.932±0.033^b^		48 h	7.871±0.384^a^	8.4±0.351^a^	10.06±0.48^b^
	72 h	1.879±0.066	1.964±0.019	2.01±0.05		72 h	7.484±0.447^a^	8.778±0.057^b^	8.84±0.353^b^
	96 h	1.949±0.015^a^	2.005±0.006^b^	1.973±0.016^ab^		96 h	7.687±0.42^a^	8.382±0.193^a^	9.83±0.197^b^

The increase of total free amino acids was due to specific changes in arginine, proline, glycine, alanine, taurine, serine and glutamic acid. Arginine, proline and alanine showed the highest proportions of total free amino acids, and all exceeded 1 mg/g wet weight. The arginine content was the highest, accounting for ∼20% of the total free amino acid content. The arginine content at 16‰ salinity was significantly higher than the controls at 6 h and 96 h (*P*<0.05), but no significant changes were observed at 12 h to 72 h. The arginine content at 30‰ salinity did not significantly change from 6 h to 96 h, except for the 48 h (*P*<0.05) time point. The proline content accounted for ∼17% of the total free amino acid content and tended to increase from 6 h to 96 h. Compared with the control and 16‰ treatment groups, exposure to 30% salinity resulted in significantly higher values (*P*<0.05) at all times points, except for 12 h and 72 h. The alanine content accounted for ∼14%, and the alanine content at 30‰ salinity was significantly lower than the control at 12 h (*P*<0.05). The alanine content at both 16‰ and 30‰ salinity increased significantly from 24 h to 96 h. Glycine and glutamic acid also significantly increased after 6 h of acclimation (*P*<0.05) but declined to the control level and remained constant from 12 h to 96 h.

## Discussion

The glutamate dehydrogenase (GDH) reaction controls amino acid metabolism in metazoans [21]. In this study, we cloned a GDH gene in *E. sinensis* that encoded a 564 amino acid protein, and this protein was also conserved in other species. As indicated by the mitochondrial localization of this enzyme, its coding sequence included an N-terminal mitochondrial signal sequence peptide, which is in agreement the finding from Li et al. (2009) in *L. vannamei* [26]. This is the first time that the GDH gene has been identified from a species in Brachyura of Decapoda. GDH is usually divided into four classes: GDH-1 and GDH-2 are small hexameric enzymes with a broad taxonomic distribution for ammonia assimilation [28]–[30], GDH-3 is a class of larger GDH only found in fungi and protists, which functions in glutamate catabolism [31], and GDH-4 is only known to be present in eubacteria [30]. McDaniel et al. (1986) found that bovine heart GDH is composed of two isozymes, but no information related to the gene encoding the two isozymes has been reported until recently [32]. In crustaceans, only one GDH cDNA was found in *T. californicus* [21], and two GDH cDNAs, GDH A (a truncated gene) and GDH B, were found in *L. vannamei* [26]. In this study, only one GDH was identified in *E. sinensis*.

Here, we successfully determined the target gene expression using specific primers and qPCR. We found different GDH expression profiles in all of the tissues examined. The GDH gene was expressed mainly in the muscle of *E. sinensis*, which is in agreement with previous findings in *L. vannamei* [26]. The localized expression of GDH can be explained by its function in the metabolism of both alanine and proline [21], [33]. Muscle is the major tissue for protein deposition and possibly represents the main pool of amino acids. The metabolism of most amino acids also occurs in this tissue. Therefore, the pool of amino acids could be mobilized when free amino acids are required for a physiological function [26]. Following a salinity change, the loss of free amino acids from the muscle will result in the release of amino acids into the blood, and the additional osmotic load at the blood level will increase the inward water flow from the external medium [34]. Free amino acids are involved in osmoregulation in crustaceans and contribute to the osmoregulation capacity during ambient salinity changes [35]–[37]. Therefore, our results, together with the finding in *L. vannamei* [26], indicate that muscle should be the optimal site for the gene expressions of several key enzymes in the osmolyte metabolism of crustacean species.

In this study, GDH gene expression was significantly increased in the muscle of *E. sinensis* following transfer from freshwater to water with 16‰ and 30‰ salinity. Additionally, the rate and extent of GDH gene expression correlated with the level of salinity. GDH has been implicated as a potential control point for amino acid synthesis. However, research on the effect salinity on GDH gene expression is very limited and controversial [38]. Li et al. (2011) found that the GDH expression profile of *L. vannamei* was similar to that of the Na^+^-K^+^ ATPase, which is a proven a marker of osmoregulation capacity [27]. Thus, GDH activity might be a practical indicator reflecting the osmoregulation capacity by regulating the synthesis of alanine and proline [33]. This result is consistent with the findings of Arena et al. (unpublished data), which demonstrated that increased GDH activity is concomitant with the ability to adapt to salinity and that the GDH expression escalates in response to high protein in the *L. vannamei* diet. However, in *T. californicus*, GDH transcription and enzyme activity did not appear to function in the regulation of alanine and proline accumulation under hyperosmotic stress [21], [39], [40]. The conflicting results between *T. californicus*, *E. sinensis* and *L. vannamei* can be explained by the differences in species and experimental design. For example, *T. californicus* is a small crustacean species, and Willet and Burton (2003) used the whole organism to quantify GDH expression. *T. californicus* were transferred from 50% to 100% seawater for periods of time ranging from 5 to 90 min [21]. Alternatively, in this study, *E. sinensis* is a macro-crustacean species, only the dominant tissue (muscle) was used to investigate GDH expression, and the salinity stress time was measured over 96 h to allow GDH to have adequate time to respond to the ambient salinity challenge. Our results, together with the findings in *L. vannamei*, indicate that GDH is a key enzyme that plays an important role in osmoregulation in macro-crustacean species.

Because crustaceans exhibit a variety of osmotic and ionic regulatory mechanisms and free amino acids play important roles in the process of osmoregulation in crustaceans [41], the muscle free amino acid contents were analyzed. The total, essential and non-essential free amino acids contents in the muscle of crabs increased significantly after transfer to salt water, and the values at 30‰ salinity were higher than those at 16‰ salinity after 24 h. These data suggest that *E. sinensis* could rapidly improve the free amino acid concentration in the body to regulate hemolymph osmotic pressure under hyperosmotic stress by mobilizing the amino acids pool of muscle, and crabs require a relatively longer time and more free amino acids at higher salinity. However, total free amino acid content at 30‰ salinity was significantly reduced at 12 h. Following *E. sinensis* adaptation to seawater, the amino acids were more concentrated in the muscle than in the blood [20]. Hemolymph free amino acids are directly involved in mediating the response to salinity exposure. Under high salinities, total free amino acid concentrations in *Macrobrachium rosenbergii* hemolymph increased 2.5-fold compared to original values [42]. Meanwhile, the muscle is the main amino acid pool, which plays a role in storing and supplying amino acids to the hemolymph. The loss of free amino acids from the muscle will result in the release of amino acids into the blood [34], which serves to leads to the high free amino acid concentration in the hemolymph that is observed under high salinity stress (30‰), while showing a short-term reduction at 12 h.

Free amino acid concentrations increased sharply when *M. rosenbergii* was adapted to high salinity, and these increases were due to specific changes in glycine, arginine, alanine, proline and lysine, among which alanine showed the greatest increases [42]. In *Callinectes sapidus*, proline was primarily responsible for the increase of free amino acids at higher salinity, indicating that the induction of proline synthesis is regulated by the synthesis of one of the enzymes catalyzing the three steps in the glutamate to proline pathway or a protein acting to stimulate the activity of one of those enzymes [43]. In this study, the muscle free amino acid concentration increase was due to specific changes in arginine, proline, glycine, alanine, taurine, serine, and glutamic acid, with arginine, proline and alanine, accounting the highest proportions of total free amino acids. Arginine, proline and alanine increased significantly with increasing salinity and time. The increased GDH expression in *E. sinensis* correlated with the increase of proline and alanine in this study, revealing the important role of GDH in the synthesis of glutamate acid, proline and alanine, which improved hemolymph osmotic pressure. It would be interesting to compare the respective roles of GDH and the Na+-K+ ATPase in *E. sinensis*, which presented one of the highest degrees of adaptation to salinity in the posterior gills, similar to *C. sapidus* [44]. Therefore, further study on this topic should be conducted.

In conclusion, a full-length cDNA sequence of GDH from *E. sinensis* was obtained and characterized in this study. GDH is primarily expressed in the muscle of *E. sinensis* relative to other tissues. Thus, acute hyperosmotic challenge enhances GDH expression in the muscle of *E. sinensis* to accelerate free amino acid metabolism, especially the synthesis of proline and alanine by increasing the synthesis of glutamic acid, to meet the demand for osmoregulation.

## Materials and Methods

### Animals

Healthy male *E. sinensis* weighing 109–121 g were obtained from a farm in the district of Shanghai, China. The crabs were acclimated in aquariums for 10 d with fully aerated fresh water, and the temperature was maintained at 15–15.5°C. After 10 d, 144 healthy crabs were divided into three salinity groups (0, 16 and 30‰) in aquariums (80 cm×45 cm×58 cm). Three aquariums were maintained at each salinity treatment, and 16 crabs were housed in each aquarium. Muscle, hepatopancreas, gill, hemocyte, heart, intestine and thoracic ganglia from six healthy crabs were sampled for tissue specific expression of the GDH gene, and muscle was used for GDH cDNA cloning. Muscle from six crabs in each treatment group was sampled at 0, 6, 12, 24, 48, 72 and 96 h for free amino acids analysis and GDH expression changes. The crabs were not fed during the challenge test, and all crab specimens were frozen rapidly in liquid nitrogen and transferred to −80°C for storage prior to RNA extraction.

### Total RNA Extraction

Total RNA was extracted from the target tissues using a Unizol Reagent Kit (Biostar, Shanghai, China) according to the manufacturer's protocol. The concentration and quality of total RNA were estimated by spectrophotometry (absorbance at 260 nm and 280 nm) and agarose-gel electrophoresis, respectively.

### RACE ready cDNA reverse transcription

Total RNA was reverse transcribed using the PrimeScript™ RT reagent Kit (TaKaRa, Dalian, China) for real-time quantitative RT-PCR (qRT-PCR) analysis. The reactions were carried out in a total volume of 10 µl, and the volume of each reaction component was as follows: 2 µl of 5×PrimeScript™ Buffer, 0.5 µl of random 6 mers (100 µM), less than 500 ng of Total RNA and up to 10 µl of RNase Free dH_2_O. The reverse transcription was conducted at 37°C for 15 min and 85°C for 5 seconds. RACE-Ready cDNA was generated by using the SMARTer™ RACE cDNA Amplification Kit (Clonetech, USA) with 3′-CDS Primer A (Table 2) and 5′-CDS Primer A (Table 2) for 3′-RACE and 5′-RACE, respectively. The reaction components and conditions were performed according to the manufacturer's recommendations.

**Table 2:** Sequences of primers.

Primers	Sequences (5′-3′)
cDNA cloning	
GDH 5′ primer (GSP1)	CGGCGTTGCCCTTGTGAATGACC
GDH 3′ primer (GSP2)	TGAGAAGGTCATTCACAAGGGCAACG
3′-CDS Primer A	AAGCAGTGGTATCAACGCAGAGTAC(T)30VN (N = A, C, G, or T; V = A, G, or C)
5′-CDS Primer A	(T)25VN (N = A, C, G, or T; V = A, G, or C)
UPM-Long	CTAATACGACTCACTATAGGGCAAGCAGTGGTATCAACGCAGAGT
UPM-Short	CTAATACGACTCACTATAGGGC
RT-PCR and real-time PCR analysis	
GDH-RT1	TTCATCGTTCAGGGCTTTGG
GDH-RT1R	GGTGCCATTTTCAATCTTCCAG
β-actin RT2	GCATCCACGAGACCACTTACA
β-actin RT2R	CTCCTGCTTGCTGATCCACATC

### The cloning of full-length GDH cDNA

The full-length cDNA sequence of the GDH gene was obtained by using the SMART™ RACE cDNA amplification kit (Clonetech, USA). First, two degenerate primers (GDH3 and GDH2R) were designed to amplify a cDNA fragment, and then two gene-specific primers, GDH 5′ primer (GSP1) and GDH 3′ primer (GSP2) (Table 2), were designed based on the partial cDNA sequence of GDH from *E. sinensis* (accession number GU219830) to obtain the complete cDNA sequence. The GDH 5′ primer and the universal primer A mix (UPM) were used in a PCR reaction for 5′-RACE, and the GDH 3′ primer and the universal primer A mix (UPM) were used in a PCR reaction for 3′-RACE. The PCR reactions were performed in a total volume of 25 µl containing 2.5 µl of 10×Ex Hot Star buffer, 2.0 µl of dNTP mix (2.5 mM each), 2.5 µl of 10×UPM, 0.5 µl of each primer (10 uM), 15.37 µl of double-distilled water, 0.13 µl of Ex Hot Star Taq polymerase (TaKaRa, Dalian, China), and 2 µl of cDNA template. The PCR conditions were as follows: 5 cycles of 94°C for 30 s, 72°C for 3 min; 5 cycles of 94°C for 30 s, 70°C for 30 s, and 72°C for 3 min; 25 cycles of 94°C for 30 s, 68°C for 30 s and 72°C for 3 min. The PCR products were excised from the agarose gel, purified and sequenced by Sangon Biotech Company (Shanghai, China). The sequences were edited and transferred to corresponding amino acids sequence using BIOEDIT (version 5.0.9).

### Sequence analysis

Homology searches of nucleotide and amino acid sequences were conducted using the BLAST algorithm at the National Center for Biotechnology Information (NCBI) (http://www.ncbi.nlm.gov/blast). GDH cDNA and the deduced amino acid sequences from *E. sinensis* and representative vertebrates and invertebrates were compared by multiple sequence alignment using the Clustal X. SignalP 3.0 program to predict the presence and location of the signal peptide and the cleavage sites in amino acid sequences (http://www.cbs.dtu.dk/services/SignalP/). MEGA version 4.0 was used to align the cDNA sequence of *E. sinensis* with other species and construct the phylogenetic tree via Neighbor-Joining. The sequence of GDH cDNA of *E. sinensis* has been submitted to GenBank (JN628041).

### Quantitative real-time PCR (qPCR) analysis

The expression of the GDH gene in crab tissues was detected by qRT-PCR according to the full-length cDNA sequence. A pair of gene-specific primers (GDH-RT1 and GDH-RT1R) was designed to amplify a 162 bp fragment, and primers against β-actin RT2 and β-actin RT2R were used as the internal standard gene control. qPCR was carried out in the CFX96™ Real-Time System (Bio-Rad) using SYBR Green. The samples were run in triplicate and normalized to the control gene, β-actin, and the GDH expression levels were calculated by the 2^—ΔΔCt^ comparative Ct method [45]. The amplifications were performed in a 96-well plate in reaction volume of 25 µl, containing 12.5 µl of SYBR Green Premix Ex Taq™ (2×) (TaKaRa, Dalian, China), 0.5 µl (each) of gene-specific forward and reverse primers (10 µM), 2 µl of diluted cDNA template and 9.5 µl of dH_2_O. The PCR conditions were as follows: 95°C for 30 s; 40 cycles of 94°C for 15 s, 58°C for 20 s, 72°C for 20 s, and a 0.5°C/5 s incremental increase from 60 to 95°C. The results and data were analyzed using the CFX Manager™ software (Version1.0).

### Muscle free amino acids analysis

Muscle samples weighing approximately 0.15 g were taken from crabs in each treatment group about 0 and fully homogenized in the Ultrasonic Cell Disruption System by adding 3% sulfosalicylic acid with a quality volume (w/v) ratio of 1∶10. The homogenate was centrifuged (Eppendorf 5804R) twice at 12000 rpm for 20 min to precipitate protein and cellular debris. The supernatants were transferred quickly to fresh Eppendorf tubes and filtered using a 0.22 µm filter membrane. The filtered samples were then analyzed using a SYKAM S-433D amino acid analyzer to assay the free amino acid content in the muscle. Three samples were tested for each treatment group.

### Data analysis

The data were analyzed by using SPSS software (Ver17.0) and presented as the mean ± standard error (S.E.). Differences in gene expression between tissues were determined by one-way ANOVA. If a significant difference was identified, differences between means were compared by LSD's multiple range test. Differences in gene expression between the control and salinity challenged groups were determined by T-test. The level of significant and extremely significant differences were set at *P*<0.05 and *P*<0.01, respectively.
